# Medication use and treatment patterns in children and adolescents with chronic tic disorders: an EMTICS study

**DOI:** 10.1007/s00787-026-03120-5

**Published:** 2026-07-17

**Authors:** Orit Peled, Daphna Shoham, Tamar Steinberg, Alan Apter, Dana Feldman-Sadeh, Miri Carmel, Idan Barak, Zsanett Tarnok, Peter Nagy, Abraham Weizman, Pieter J. Hoekstra, Andrea Dietrich, the EMTICS collaborative group, Alan Apter, Juliane Ball, Noa Benaroya-Milshtein, Judith Buse, Francesco Cardona, Marta Correa Vela, Andrea Dietrich, Carolin Fremer, Blanca Garcia-Delgar, Tammy J. Hedderly, Isobel Heyman, Pieter J. Hoekstra, Chaim Huijser, Marcos Madruga Garrido, Davide Martino, Pablo Mir, Astrid Morer, Norbert Müller, Kirsten Müller-Vah, Alexander Münchau, Peter Nagy, Kerstin J. Plessen, Cesare Porcelli, Renata Rizzo, Veit Roessner, Daphna Ruhrman, Anette Schrag, Tamar Steinberg, Zsanett Tarnok, Frank Visscher, Susanne Walitza, Elif Weidinger, Noa Benaroya-Milshtein

**Affiliations:** 1 Department of Pharmacy, Schneider Children’s Medical Center of Israel, Petah Tikvah, Petah Tikvah, Israel; 2 Adelson School of Medicine, Ariel University, Ariel, Israel; 3 Department of Child and Adolescent Psychiatry, Schneider Children’s Medical Center of Israel, Petah Tikvah, 4920235 Israel; 4 Gray Faculty of Medical & Health Sciences, Tel Aviv University, Tel Aviv, Israel; 5 Institute of Child Neurology, Schneider Children’s Medical Center of Israel, Petach Tikva, Israel; 6 Research Unit, Geha Mental Health Center, and Laboratory of Molecular and Biological Psychiatry, Felsenstein Medical Research Center, Petah Tikvah, Israel; 7 Vadaskert Child and Adolescent Psychiatric Hospital, Budapest, Hungary; 8 Bethesda Children’s Hospital, Budapest, Hungary; 9 Department of Psychiatry, University Medical Center Groningen, University of Groningen, Groningen, The Netherlands; 10 Accare Child Study Center, Groningen, The Netherlands

**Keywords:** Psychopharmacology, Tourette syndrome, Tic disorders, Attention deficit disorder (ADHD), Medications

## Abstract

The treatment of tic disorders in children and adolescents poses significant challenges, especially when comorbid conditions such as obsessive-compulsive disorder (OCD) and attention-deficit/hyperactivity disorder (ADHD) are present. Pharmacological treatments are often guided by clinical judgment, prescriber preferences, and medication availability. This study describes pharmacological treatment use in a large cohort of children and adolescents with chronic tic disorders (CTDs). We analyzed baseline data from the European Multicenter Tics in Children Study (EMTICS) to examine associations between medication use, tic severity, and psychiatric comorbidities, using parent-reported questionnaires and clinical interviews to assess tics, obsessive-compulsive disorder (OCD), attention-deficit/hyperactivity disorder (ADHD), oppositional defiant disorder (ODD), and children’s self-rated premonitory urges. Among 709 children aged 3–16 years with CTDs, 230 (32%) received any pharmacological treatment, 22% received antipsychotics and 10% received ADHD medications, with limited use of alpha-2 agonists (3%). Medication use was more common among individuals with comorbid ADHD (54%) than those with CTD only (23%) or comorbid OCD only (26%). Those on tic-suppressing medications had significantly greater severity of tics, tic-related impairment, and comorbid ADHD, ODD, and obsessive symptom severity compared to those with non-tic-suppressing medications. There was no relationship with the severity of compulsions or premonitory urges. While largely consistent with published guidelines, these findings indicate limited utilization of recommended agents, such as alpha-2 agonists, alongside higher reported symptom severity among those receiving tic-suppressing medications. Further studies should explore the integration of pharmacological, behavioral, and psychoeducational approaches tailored to comorbidity profiles and symptom severity.

**Supplementary Information:**

The online version contains supplementary material available at https://doi.org/10.1007/s00787-026-03120-5.

## Introduction

Tic disorders are grouped under the neurodevelopmental disorders and are the most common movement disorder in children, with a prevalence of approximately 1–3% of children [1]. Tics are sudden, rapid, recurrent, nonrhythmic movements or vocalizations [2], which are usually preceded by an uncomfortable physical sensation, i.e., a premonitory urge [3]. In children, tics appear most frequently as part of a chronic tic disorder (CTD), including Tourette syndrome (TS) [4], which is characterized by the presence of at least two motor and one vocal tic [5]. Tic disorders are frequently accompanied by psychiatric comorbidities, which often serve as the primary motivation for seeking treatment^6^. The most prevalent psychiatric conditions observed along with tic disorders include attention deficit hyperactivity disorder (ADHD), obsessive-compulsive disorder (OCD), anxiety and depressive disorders, autism spectrum disorder (ASD), and disruptive behavior disorders (e.g., oppositional defiant disorder [ODD]) [6]. A thorough evaluation of a patient with a tic disorder is essential for determining the most suitable treatment approach. This evaluation encompasses an examination of psychiatric comorbidities as well as the physical, emotional, and social impacts of the disorder [2]. When quality of life is impaired, there are established treatment options that may be considered [1].

According to the clinical guidelines of the European Society for the Study of Tourette Syndrome (ESSTS), priority is given to psychoeducation and behavioral therapy, which strengthens the patient’s self-regulation and, in turn, their autonomy [7]. When these treatments prove ineffective or are not available or feasible (e.g., due to lack of motivation or young age) pharmacological treatment strategies can be considered. In addition, pharmacological treatment is more readily initiated and typically yields effects within days, whereas behavioral therapy often requires several weeks. Pharmacological treatment may thus be preferred when rapid symptom reduction is warranted [8, 9].

The primary objective of drug therapy is to diminish tics and/or to address concurrent disorders, most notably ADHD and OCD [10]; Research on pharmacological treatments for tic disorders in children and adolescents is constrained by limited empirical data. Evidence from randomized controlled trials is scarce, with only a few high-quality studies available, and the tic-suppressing effectiveness of most agents remains uncertain [11, 12]. Furthermore, the risk of side effects may surpass therapeutic benefits. With a lack of robust guidance on the drug of choice and unclear evidence of efficacy, the treatment of tics mostly relies on clinical experience, the preferences of the individual prescriber and patient preference, and availability [7, 11, 12]. Multiple types of medications can be found in the armamentarium for the psychiatrist coming to treat tic disorders and related comorbidities. The treatment of choice should be patient-individualized, decided according to the compound’s mechanism of action, mostly corresponding to the main complaint: tics and/or comorbidities. Besides, as a result of the uncertain therapeutic benefits weighing against the potential side effect profile, tic-suppressing medications are typically prescribed for patients whose tics cause marked distress or functional impairment, whereas individuals with milder symptoms may remain untreated or receive other psychotropic agents [7]. Accordingly, in already treated participants at a single time point, greater tic severity would be expected among those using tic-suppressing medications compared to those who are untreated or receiving other psychotropic agents.

Systematic reviews and guidelines were recently published by the American Academy of Neurology (AAN) [11, 12] and ESSTS [7]. According to these guidelines, antipsychotics are the pharmacological treatment of choice for tic reduction, their effectiveness most likely being due to their anti-dopaminergic properties. Aripiprazole, a second-generation antipsychotic, has become the most frequently used agent for the treatment of tics due to its good effectiveness on tics and favorable profile of adverse effects [7, 10, 13]. Premonitory urges closely correlate with tic frequency and intensity and represent a core feature of tic phenomenology, providing additional insight into symptom expression among treated youth. Subjects with a tic disorder and comorbid ADHD may benefit from the use of noradrenergic agents, also known as alpha-2 adrenergic receptor agonists, such as clonidine and guanfacine, in reducing tics and improving ADHD symptoms [7, 11, 12]. For co-existing OCD, first-line treatment is cognitive behavioral therapy (CBT) and/or selective serotonin reuptake inhibitors (SSRIs), with antipsychotic augmentation considered only when symptoms remain refractory [7]. Of note, only a few medications used for the treatment of tics are currently regulatorily approved. Aripiprazole, haloperidol, and pimozide are approved by the Food and Drug Administration (FDA) for the suppression of tics/TS, while in Europe, only haloperidol holds a licensed indication for tic disorders. Therefore, most medications are prescribed off-label as tic suppressors [7, 10]. Describing real-world patterns of medication use may provide insight into their alignment with recommended practices.

Despite consensus guidelines for the pharmacological treatment of chronic tic disorders (CTDs) in children and adolescents, the evidence base describing how these guidelines are actually translated into prescribing practice remains limited and geographically fragmented. Existing pharmacoepidemiologic data on pediatric CTDs derive predominantly from single-country administrative claims registries [1, 4, 10, 14] or from single-site clinical samples. These data sources, while informative, are constrained in two important ways: administrative datasets lack standardized clinician-rated symptom severity, and single-site clinical samples cannot capture cross-national variability in prescribing culture, regulatory approvals, and reimbursement. As a result, no harmonized European benchmark currently exists that links real-world medication use to clinician-rated tic severity and to detailed comorbidity profiles in the same pediatric cohort.

The present study addresses this gap. Using the baseline dataset of the European Multicenter Tics in Children Study (EMTICS), a large prospectively recruited, multi-country European cohort of 709 children and adolescents with CTDs, phenotyped with standardized clinician-administered instruments, we are able to link real-world prescribing patterns to clinician-rated symptom severity across nine European countries. This design makes it possible to identify specific care gaps, such as the underutilization of alpha-2 agonists during the study period, that would not be visible in single-country data.

The primary aim of our current study therefore was to describe the use of pharmacological treatment among children and adolescents with a chronic tic disorder who participated in the longitudinal EMTICS cohort study [15]. Our investigation focused on medication use (i.e., tic-suppressing and other psychotropic medications) across comorbid diagnoses (i.e., CTD with/without comorbidity) and the relationship with severity scores of tics, premonitory urges and comorbid symptoms. We focused on ADHD and OCD as these comorbidities are most directly linked to pharmacological decision making in tic disorders, and included ODD due to its close association with ADHD. We hypothesized that the severity of tics, premonitory urges and comorbid symptoms would be more severe in children and adolescents receiving tic-suppressing medications than in those who were not treated with such medications. Furthermore, we expected higher medication use in adolescents than in children [16], and differences in symptom severity profiles across different medication groups (i.e., tic-suppressing, ADHD, combined, or non/all other psychotropic treatments).

## Methods

### Participants

Our study sample comprised 709 children and adolescents aged 3–16 years diagnosed with TS or another CTD, according to the criteria of the *Diagnostic and Statistical Manual of Mental Disorders*, Fourth Edition, Text Revision (DSM-IV-TR [5], Table 1). The study sample participated in the baseline assessment of the longitudinal EMTICS which took place from January 2013 to October 2015 [15]. The study’s inclusion and exclusion criteria have been described in detail elsewhere [15].

**Table 1:** Demographic and clinical characteristics of children and adolescents with tics and comorbid diagnoses and symptom severity (*n* = 709)

	*N*	%	Mean ± SD	Skew	Range	%Zero**
Age	709	-	10.7 ± 2.82		3.35-17.0	
Sex (Male)	544	76.7	-		-	
Parental educational level^*a*^	685		3.98 **±** 1.22		1.00–6.00	
Ethnicity (Caucasian)	601	84.8			-	
Tic Disorders						
Tourette’s Disorder	624	88.0				
Chronic Motor Tic Disorder	79	11.1				
Chronic Vocal Tic Disorder	6	0.9				
Tic Severity						
Motor tics^*b*^			12.3 **±** 4.63	-0.39*	0–23	3.1
Vocal tics^*b*^			7.27 **±** 5.56	0.25*	0–22	25.2
Total tic severity^*b*^			19.6 **±** 8.64	0.15	0–44	1.6
Tic impairment^*b*^			14.5 **±** 11.9	0.59*	0–50	24.3
ADHD diagnosis (total)^*cd*^	195	27.5				
ADHD inattentive type^*cd*^	77	10.9	10.7 **±** 7.01	-0.39*	0–27	4.7
ADHD hyperactive-impulsive type^*cd*^	13	1.8	8.89 **±** 6.54	0.64*	0–27	7.8
ADHD combined type^*cd*^	105	14.8	19.5 **±** 12.4	0.53*	0–54	1.8
OCD^*ce*^	296	42.0				
Obsessions^*e*^			2.92 **±** 4.63	1.33*	0–18	65.2
Compulsions^*e*^			3.65 **±** 4.98	1.01*	0–20	57.2
Total OCD severity score^*e*^			6.58 **±** 8.63	1.17*	0–36	51.5
ODD^*cd*^	87	12.3	7.97 **±** 5.88	0.69*	0–24	7.9
Premonitory urges^*f*^	542	76.4	17.6 **±** 5.88	0.22*	9–34	0.0

Data are given as mean ± standard deviation (range) for continuous variables and number (percentage) for categorical variables. *Skewed (*p*<.05) clinical measures were based on the skewness over its standard error. **0% relates to percent diagnosed without the specific symptom

Abbreviations:*ADHD*Attention Deficit Hyperactive Disorder, *OCD* Obsessive Compulsive Disorder, *ODD* Oppositional Defiant Disorder, *PUTS* Premonitory Urge for Tics Scale.^a^Parental education level, as a proxy for socio-economic status, was recorded using a 6-point scale (1 = 7 years of schooling, 2 = junior high school, 3 = high school diploma, 4 = associate degree, 5 = bachelor degree, and 6 = post graduate/graduate/professional degree).^b^*YGTSS*Yale Global Tic Severity Scale,^c^Comorbid clinical diagnoses were defined using DSM-IV-TR criteria for ADHD and OCD (including clinical cut-off scores used for OCD CY-BOCS total scores ≥ 16) and ODD (≥ 1.88 on the ODD SNAP-IV),^d^severity scores based on the *SNAP-IV*Swanson, Nolan and Pelham- IV 26-item rating scale,^f^*PUTS* Premonitory Urge for Tics Scale

### Procedures

Written informed consent was obtained from parents/guardians and assent or consent from adolescents, per local ethical regulations. The study was approved by all local ethics committees. This analysis used parent-rated or self-administered questionnaires to assess premonitory urges, and ADHD, ODD, and OCD symptom severity, and a clinical interview [15]. Clinicians confirmed the diagnosis of TS/CTD and evaluated comorbid psychopathology of ADHD and OCD per DSM-IV-TR criteria [5]. For cases of diagnostic uncertainty, we used consensus scoring by an expert team at each clinical center. Clinicians also rated tic and OCD severity using validated measures.

### Clinical measures

Demographic and clinical data included the child’s sex, age at evaluation, ethnicity, and psychotropic medication use at baseline. Parental education, used as a proxy for socioeconomic status, was rated on a 6-point scale (1 = 7 years of schooling; 2 = junior high; 3 = high school diploma; 4 = associate degree; 5 = bachelor’s degree; 6 = postgraduate/professional degree), and averaged across both parents. Symptom severity was obtained for tics, comorbidities (OCD and ADHD, ), and premonitory urges, using the following scales:

#### Tics

Yale Global Tic Severity Scale (YGTSS) Tic severity was assessed using the YGTSS, a semi-structured, clinician-rated gold standard for evaluating tic severity and frequency in children and adults [17, 18]. Internal consistency in current study: Cronbach’s alpha and McDonald’s Omega are α = 0.87 and Ω = 0.58 respectively (see Haas et al., 2021) [19]. Scores included: motor tics (0–25, α = 0.89), vocal tics (0–25, α = 0.85), and total tic severity (0–50, α = 0.87, Ω = 0.58), and tic impairment (0–50, α = 0.88, Ω = 0.56).

#### OCD

The Children’s Yale-Brown Obsessive-Compulsive Scale (CY-BOCS) is a clinician-administered semi-structured interview assessing OCD severity in children [20–22]. It yields total (0–40, α = 0.93 in current study), obsession (0–20; α = 0.92 in current study), and compulsion (0–20; α = 0.94 in current study) severity scores. Dichotomous OCD diagnosis was defined by DSM-IV-TR criteria and/or CY-BOCS total score ≥ 16, a commonly used clinical cutoff [20, 21, 23].

#### ADHD and ODD

The Swanson, Nolan, and Pelham, version IV (SNAP-IV) is a validated 26-item parent-rated questionnaire assessing ADHD and ODD symptoms (α = 0.95, in current study) [24, 25]. ADHD severity was measured using the first 18 items (0–54; Inattention: 0–27, α = 0.91; Hyperactivity-Impulsivity: 0–27, α = 0.90; total α = 0.94), and ODD severity using the last 8 items (0–24, α = 0.91). ADHD diagnosis followed DSM-IV-TR types; ODD diagnosis was defined by a mean score ≥ 1.88 on the ODD scale [24–26].

ASD symptoms were assessed using The Autism Spectrum Screening Questionnaire (ASSQ) [27]. A three-point scale was used to assess symptoms across four domains (social interaction, communication, restricted and repetitive behavior, motor clumsiness and other associated symptoms), with a higher score indicating higher ASD symptom severity (27 items, range 0–54, α = 0.90). A Dichotomous ASD diagnosis was defined as scoring above known cut-off (ASSQ score ≥ 19) [27].

#### Premonitory Urges

The Premonitory Urge for Tics Scale (PUTS) was used to assess the severity of premonitory urges (child self-rated) [3, 28]. Item 10 (measuring the ability to stop tics briefly) was excluded from further analyses due to its lower correlation with the rest of the scale across age groups [3, 28, 29]. Thus, our operational total score range for this instrument is 9–36. A total PUTS severity score above 9 was defined as indicative of the dichotomous experience of premonitory urges sensation for tics [28]. Furthermore, a higher total score indicated more severe premonitory urges, with increasing scores corresponding to a greater intensity of the urges experienced. Cronbach’s alpha in our study was α = 0.84.

### Assessment of medication use

Psychotropic medication use in the previous two weeks was recorded by the parents on a pre-specified listing in the paper questionnaire booklet and confirmed by the study clinician during the first clinic visit, with the possibility of adding additional psychotropic medications. We defined tic-suppressing medications as first and second-generation antipsychotics, and included alpha-2 agonists (suggested for both the treatment of tics and ADHD; note that the predefined medication listing did not include guanfacine). The medications for ADHD consist of medications exclusively indicated for ADHD (i.e., stimulants and non-stimulant), whereas medications used for OCD (e.g., SRIs and benzodiazepines) also have other indications, such as anxiety or depression. See Supplementary Table S1 for the listing of individual medications.

### Statistical analyses

#### Preliminary considerations and organization of the data for analyses

##### Imputed missing data

A preliminary analysis performed on variables related to tics and comorbidities, using Little’s MCAR test indicated that data were missing completely at random (χ²=112.26, df = 109, *p*=.396). Notably, missingness on all primary outcome variables, tic severity (YGTSS motor and vocal tic counts and total scores), OCD severity (CY-BOCS), and ADHD/ODD symptoms (SNAP-IV) was zero. Missingness was confined to covariates and to the PUTS measure, at very low rates (≤ 3.4% on covariates; 6% on PUTS). Under these conditions, complete outcome data and low-rate MCAR covariate missingness, single imputation has been shown to yield estimates and inferences essentially equivalent to multiple imputation (Enders 2010) [30]. A single round of imputation was therefore performed using SPSS v29 [30], using random substitution based on known correlated variables. To address the undere stimation of standard errors, we used Generalized Linear Models with robust (sandwich) standard errors throughout (see Analytical Strategy below), independent of the imputation procedure.

##### Normality and skewness testing

 We examined histograms, Q–Q plots, and skewness/kurtosis statistics (Table 1; asterisks denote p < .05 departures from normality/symmetry). Accordingly, we modeled skewed, strictly positive scales with Gamma GLMs (log link) and retained Normal GLMs (identity link) only where variables supported approximate normality, specifically, YGTSS motor and total tic severity (see Supplementary, Table S2 & S3). Because vocal tics exhibited substantial zero inflation, they were analyzed with a two-part model (any vs. none; then analysis of positive values).

##### Diagnostic groupings

We defined four diagnostic groupings, i.e., CTD-only (n = 326), CTD+OCD (n = 188), CTD+ADHD (n = 87), and CTD+OCD+ADHD (n = 108).

##### **Medication groupings**

Two grouping approaches were defined: (1) Binary split: Group 1 (G1): use of tic-suppressing medications (first/second generation antipsychotics, and/or alpha-2 agonists) vs. Group 0 (G0): no use of tic-suppressing medications (all other medications). (2) Four-group classification based on three binary indicators: Group A: tic-suppressing medications only; Group B: combined tic-suppressing and ADHD medications (i.e., stimulants and non-stimulant); Group C: ADHD medications only; and Group D: other, i.e., neither tic suppressing medication, nor ADHD medication, but could include the use of other psychotropic drugs (e.g., antidepressants: *n* = 24; benzodiazepines: *n* = 12; propranolol, *n* = 1; note that the low prevalence did not allow for dedicated sub-analyses on these type of drugs).

##### Country effect assessment

The survey included 16 clinical centers across 9 countries, yet the center sample sizes were variable and sometimes too small for analysis (i.e., 5, 19, 24). Therefore, we modeled country-level effects rather than center-level effects, as an additional factor by regressing each outcome variable on medication use separately for each country, although we acknowledge there may still be site differences in some countries. Because several centers had small sample sizes, we modeled country-level effects rather than center-level random effects, using a country factor derived from country-specific medication–outcome associations and entering this factor as a covariate in all models. Those countries in which group difference was found to be significant were coded one, while other countries were coded zero. This process resulted in a country binary factor for each outcome measurement that was then added as an additional explanatory factor into each prediction model. Because this was a binary indicator (0/1) rather than a multi-level categorical country variable, no reference country was defined. The indicator was entered as a fixed-effect covariate in each outcome model to adjust for cross-national heterogeneity; it was not used to estimate or compare country-specific effects.

#### Analytical strategy

Two main types of analyses were conducted. The first type focused on comparing the proportions of medication use within and across diagnostic groupings with/without comorbid conditions, in order to describe differences in pharmacological treatment patterns. The second type of analyses examined whether medication groupings are associated with continuous symptom measures (severity of tics and comorbid symptoms, and premonitory urges). This approach allowed us to assess whether different medication categories are linked to variations in symptom expression and clinical presentation.

1. Comparing proportions of medication use within and between diagnostic groupings.

Usage across diagnostic groups (i.e., CTD-only, CTD + OCD, CTD+ADHD, CTD + OCD+ADHD) was compared using within-group proportions of medication use. Between-diagnostic group comparisons of medication use were conducted for three treatment groups separately: Participants treated with tic-suppressing medications only, ADHD medications only, and combined treatments (tic-suppressing and ADHD). Since comparisons of medication use were conducted, the fourth group was excluded, i.e., participants using neither tic suppressing medication, nor ADHD medications.

Proportion comparisons and ranking: To compare medication-use proportions within and between comorbid diagnosis groups, we conducted pairwise tests of two proportions with multiplicity control (Marascuillo-type multiple-comparison procedure). This method was chosen as it makes explicit where differences occur. These comparisons were adjusted for multiple comparisons using the Bonferroni correction method, where *p*<.05 was considered the pre-adjustment significance level. As an additional exploratory analysis, we conducted age-stratified comparisons of any medication use (< 12 vs. ≥ 12 years), overall and within each diagnostic/comorbidity group, using chi-square tests (and corresponding proportion comparisons where appropriate).

We present two dimensional rankings, both across comorbidity groups within a medication row (row-wise) and, where relevant, across medication categories within a comorbidity group (column-wise) to convey the two margins of the table succinctly. We report letter-based rankings (a, b, c) within each row to indicate ordered group differences: groups that share a letter do not differ after adjustment; groups with different letters do (a lowest proportion, followed by b and c).

2. Covariate-Adjusted Generalized Linear Models for tic severity comparisons between medication groupings

To examine associations between medication-use and symptom severity, we treated medication groupings as predictor variables and tic severity, comorbid psychopathology scores, and premonitory urges as continuous outcome variables. Medication group was entered according to the two grouping approaches defined above as a categorical factor (dummy treatment coding), and all models were adjusted for relevant covariates, including age, sex, parental education (1–6 scale), ethnicity, and the country factor (as defined above).

Analyses were performed using generalized linear models (GLMs), with distribution–link functions informed by diagnostic evaluation: Normal/identity links were used where distributions were approximately normal, and Gamma/log links were applied for right-skewed scales. Vocal tic severity demonstrated zero inflation; therefore, we employed a two-part model in *Mplus* (version 8.3), consisting of a logistic component (any vs. none) and a GLM applied to nonzero values.

Post-hoc contrasts were Bonferroni-adjusted, and all inferential tests used Huber–White robust (sandwich) standard errors. Because few symptom severity outcomes showed non-normal, right-skewed, or zero-inflated distributions, and because models required covariate adjustment, we analyzed data using generalized linear models (GLMs) [31]. To quantify the magnitude of group differences beyond statistical significance, we computed effect-size estimates appropriate to each analysis type. For the GLM-based continuous-outcome comparisons (Tables 3 and 4), we report Vandekar’s Robust Effect Size Index (RESI; Vandekar et al.) [32], which is derived from the Wald test statistic, is on a Cohen’s-d-like scale, and is robust to model misspecification. Magnitude is classified following the conventions of the same reference [32]. For the proportion comparisons in Table 2, we report Cohen’s h [33], which is the canonical effect-size measure for proportions; the complete set of pairwise Cohen’s h values is presented in Supplementary Table S4.

## Results

### Sample characteristics

Table 1 presents descriptive statistics for dichotomous and continuous measures of tics and comorbid psychopathology in the full sample (*N* = 709). Ages ranged from 3 to 17 years, with a mean of 10.7 years (SD = 2.8). Sex proportion was biased towards males (76.7%). TS was the most prevalent diagnosis (88.0%). The mean total tic severity score (YGTSS) was 19.6 ± 8.6, and mean tic impairment score was 14.5 ± 11.9. The two most frequent comorbid diagnoses were OCD (42.0%; 296) and ADHD (27.5%; 195). Mean total tic severity was 22.38 ± 8.65 among participants with OCD and 22.61 ± 9.29 among those with ADHD; mean tic impairment was 17.13 ± 12.36 and 18.36 ± 13.67, respectively. 135 (19.0%) of the participants met criteria for suspected ASD, with ASD mean symptom severity score of 11.36 ± 9.27.

### Usage rates of psychotropic medications within diagnostic groups

Table 2 presents psychotropic medication usage among all participants and across comorbid diagnostic groupings. The proportion (%) of children and adolescents receiving any medication in Table 2 is presented relative to the entire sample, whereas the distribution of proportions (%) within specific drug classes and treatment groupings is presented relative to the population receiving medications. Antipsychotics were the most commonly prescribed drug class (*n* = 158, 68.7% of the medicated group and 22.3% of the study sample *n* = 709), of which the most commonly prescribed type was second-generation antipsychotics. This was followed by ADHD medications (10.3% of the study sample *n* = 73), of which stimulants were most commonly used compared to non-stimulants. Additionally, 3.4% of the study sample (*n* = 24) had received antidepressant treatment, and 2.8% (*n* = 20) had received alpha-2 agonists. It should be noted that 52 participants (7.3%) were receiving multiple medications concurrently. Due to the low prevalence of antidepressant use for OCD treatment, a separate “OCD medication” group was not created, and no statistical analyses were performed for it. Overall, any medication use was lower in participants aged < 12 versus ≥ 12 years (26.4% vs. 44.7%; χ²(1) = 23.78, *p*<.001). Within diagnostic groups, the age-related difference was significant for CTD-only (36.3% vs. 18.3%; *p*<.001) and CTD with OCD (36.2% vs. 20.2%; *p*=.016), while differences within the ADHD comorbidity groups did not reach statistical significance.

**Table 2:** Rates of Psychotropic Medications use in Children and Adolescents with a Chronic Tic Disorder (CTD; EMTICS, *N* = 709, Marascuillo-type multiple-comparison procedure) by comorbid diagnoses

	All Participants*n* = 709	CTD-only *n* = 326	CTD + OCD *n* = 188	CTD+ADHD *n* = 87	CTD + OCD +ADHD *n* = 108	Group comparisons w/wocomorbidity
	No medication	479 (68%)	250 (77%)	138 (73%)	41 (47%)	50 (46%)
Any Medication	230 (32%)	76^b^ (23%)	50^a^ (27%)	46^a^ (53%)	58^b^ (54%)	CTD only = CTD+OCD+ADHD > CTD+ADHD = CTD+OCD
Antipsychotics	158 (69%)	59 (78%)	37 (74%)	27 (59%)	35 (60%)	
First generation	29 (13%)	7 (9%)	12 (24%)	3 (7%)	7 (12%)	
Second generation	129 (56%)	52 (68%)	25 (50%)	24 (52%)	28 (48%)	
ADHD medication*	73 (32%)	12 (16%)	6 (12%)	27 (59%)	28 (48%)	
Stimulants	57 (25%)	9 (12%)	6 (12%)	23 (50%)	19 (33%)	
Non-stimulants	17 (7%)	3 (4%)	0	5 (11%)	9 (16%)	
Alpha agonists	20 (9%)	6 (8%)	5 (10%)	4 (9%)	5 (9%)	
Antidepressants, SRIs	24 (10%)	5 (7%)	7 (14%)	3 (7%)	9 (16%)	
Benzodiazepines	12 (5%)	3 (4%)	4 (8%)	0	5 (9%)	
Other (propranolol only)	1 (0.4%)	0	1 (2%)	0	0	
Treatment Groups (*n* = 214)						
Tic-suppressing medications only (Tic only)**	141^,b^ (61%)	60^,b^ (79%)	37^c, b^ (76%)	18^a, a^ (39%)	26^b, b^ (45%)	CTD + OCD > CTD + OCD+ADHD > CTD+ADHD
Tic-suppressing and ADHD medications (Tic+ADHD)	34^,a^ (15%)	5^,a^ (7%)	4^a, a^ (8%)	12^b, a^ (26%)	13^b, a^ (22%)	CTD + OCD+ADHD = CTD+ADHD > CTD + OCD
ADHD medications only (ADHD only)	39^,a^ (17%)	7^,a^ (9%)	2^a, a^ (4%)	15^b, a^ (33%)	15^b, a^ (26%)	CTD + OCD+ADHD = CTD+ADHD > CTD + OCD
Treatment Group Comparisons	Tic only > Tic+ADHD = ADHD only	Tic only > Tic+ADHD = ADHD only	Tic only > Tic+ADHD = ADHD only	Tic only = Tic+ADHD = ADHD only	Tic only > Tic+ADHD = ADHD only	

Abbreviations: *CTD* Chronic Tic Disorder, *OCD* Obsessive Compulsive Disorder, *ADHD* Attention Deficit Hyperactive Disorder, *SRI* Serotonin Reuptake Inhibitors

Percentages in the ‘Any medication’ row are calculated from the entire sample (*N* = 709). Effect sizes (Cohen’s h) for pairwise proportion comparisons are presented in Supplementary Table S4

Upper panel: Percentages in all subsequent rows (individual medication classes) are calculated only among participants who received any medication (n = 230 overall), and in accordance with specific diagnostic groupings. Because patients could receive more than one medication class, these categories are not mutually exclusive

*One patient received both a stimulant and a non-stimulant ADHD medication

**Tic-suppressing medications were classified as antipsychotics and alpha2-agonists, per guideline/review evidence and prior pharmacoepidemiologic practice

Lower panel: Latin letters denote pairwise proportion rankings: The first letter compares proportions across the comorbidity-diagnostic groups within each row (CTD+OCD vs. CTD+ADHD vs. CTD+OCD+ADHD). The second letter compares proportions across the three exclusive treatment-groups (tic-suppressing medications only vs. ADHD medications only vs. both) within each column, which are also distinguished by color. Within each comparison, “a” marks the lowest proportion, followed by “b” and “c”. Shared letters indicate no significant difference between groups

Bonferroni correction method, where *p*<.05 was considered pre-adjustment significance level

The lower panel of Table 2 shows the frequencies of different diagnoses and treatment groups (i.e. tic-suppressing medication, ADHD medication, or both). It indicates that among participants using medications, 61% (*n* = 141) received tic-suppressing monotherapy, 15% (*n* = 34) were treated with a combination of tic-suppressing and ADHD medication, and 17% (*n* = 39) with ADHD medications only. Any-medication use exceeded 50% in participants with CTD+ADHD with or without OCD (54% and 53%, respectively), whereas rates were lower in CTD-only (23%) and CTD + OCD (27%). In particular, when ADHD comorbidity was present, ADHD pharmacotherapy was more common (either combined with a tic-suppressing drug or as ADHD-only treatment) compared with groups without ADHD (i.e., 22–33% versus 4–9%, respectively, see Table 2). Thus, a small proportion also receives ADHD medication in the absence of an ADHD reported diagnosis. Numbers indicate that about 40% of those with an ADHD diagnosis do not receive ADHD medication.

Tic-suppressing monotherapy was the predominant regimen in CTD-only and CTD + OCD (76–79%( versus 39–45% in CTD+ADHD and CTD+ADHD + OCD. The overall use of tic-suppressing medications was 84–86% in CTD-only and CTD + OCD, versus 65–67% in CTD+ADHD and CTD+ADHD + OCD. In participants with comorbidities treated with medication, the use of tic-suppressant drugs was lower in those with ADHD but not in those with OCD (76% < 45% < 39%).

In the CTD + OCD and CTD+ADHD + OCD groups, 14–16% of the overall medication use is explained by antidepressant use, compared with 7% in CTD-only and CTD+ADHD. No differences were detected across diagnostic groups in the relative proportions of participants receiving ADHD medication when comparing the combined (tic-suppressing+ADHD) versus ADHD-only medication categories (Table 2).

### The severity scores of tics, comorbid psychopathology, and premonitory urges among participants with or without tic-suppressing medications

**Fig. 1:**
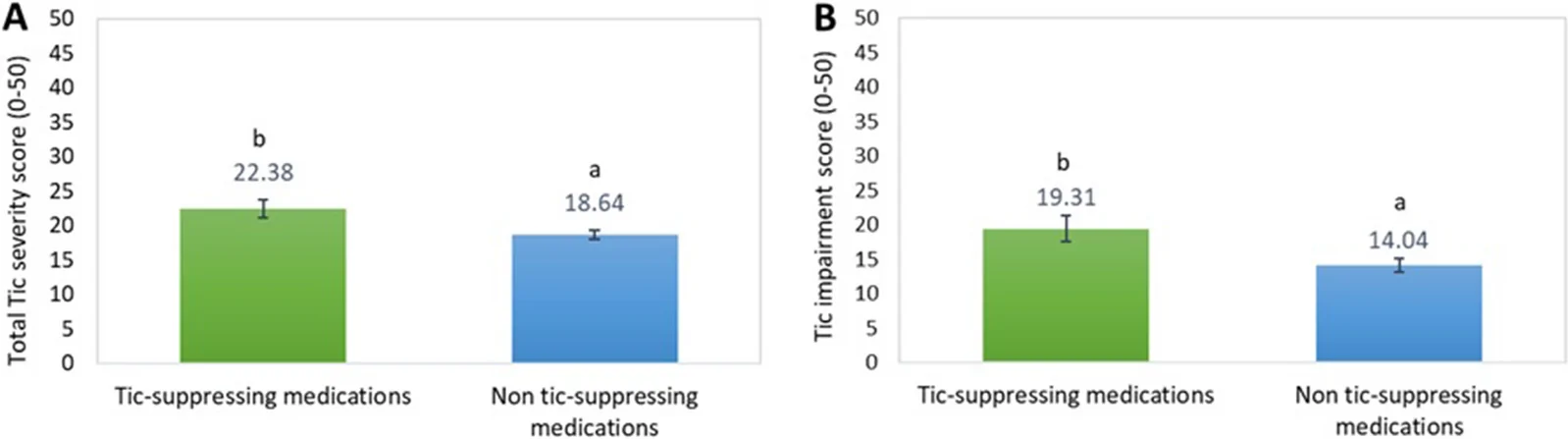
YGTSS Total Tic Severity and Impairment Scores Among Children and Adolescents with or without Tic-Suppressing Medications. This figure presents group comparisons between children and adolescents receiving tic-suppressing medications and those not receiving such treatment (i.e., receiving other medication with psychotropic activity or none) (N = 709). (**A**) Total tic severity scores and (**B**) Tic-related impairment scores, as assessed by the Yale Global Tic Severity Scale (YGTSS). Latin letters are used over each bar for statistical significance, with "a" indicating the smallest value. Data are presented as means with 95% confidence intervals (95% CI). YGTSS, Yale Global Tic Severity Scale

Table 3; Fig. 1 show group differences in continuous symptom scores, comparing children and adolescents using tic-suppressing medications with non-tic-suppressing medications (i.e., those receiving other psychotropic medications or none). Mean severity scores for all tic measures (severity and impairment), and ADHD, ODD, and obsession severity scores were significantly higher among participants using tic-suppressing medications compared to all other participants. No such differences were found for compulsions, total OCD scores, or PUTS scores.

**Table 3:** Severity Scores of Tics and Comorbidities Among Children and Adolescents with/without Tic-Suppressing Medications (*N* = 709, Covariate-Adjusted Generalized Linear Models)

Symptoms and comorbidities	Tic-suppressing medications (*n* = 175, 24.7%)		No tic-suppressing medications² (*n* = 534, 75.3%)		Wald’s χ²		RESI		Magnitude	
	Mean Score	95% CI	Mean Score	95% CI						
Tics (YGTSS¹)										
Motor tics	13.7ᵇ	13.0, 14.4	11.8ᵃ	11.5, 12.2	20.80		0.17		small	
Vocal tics	8.65ᵇ	7.81, 9.49	6.81ᵃ	6.35, 7.28	13.59		0.13		small	
Total tic severity	22.4ᵇ	21.1, 23.7	18.6ᵃ	17.9, 19.4	24.09		0.18		small	
Tic impairment	18.7ᵇ	17.0, 20.5	13.2ᵃ	12.2, 14.2	27.93		0.19		small	
ADHD symptoms (SNAP-IV¹)										
Inattention	11.9ᵇ	10.9, 12.9	10.3ᵃ	9.7, 10.8	7.49		0.10		small	
Hyperactivity	9.61ᵇ	8.66, 10.6	8.68ᵃ	8.15, 9.21	2.69		0.05		weak	
Combined	21.7ᵇ	19.9, 23.4	18.9ᵃ	17.9, 19.9	6.79		0.09		weak	
ODD symptoms (SNAP-IV ODD¹)	8.86ᵇ	7.97, 9.74	7.68ᵃ	7.18, 8.17	5.05		0.08		weak	
OCD symptoms (CY-BOCS¹)										
Obsessions	3.73ᵇ	3.05, 4.41	2.66ᵃ	2.28, 3.04	6.97		0.09		weak	
Compulsions	4.10	3.35, 4.86	3.52	3.09, 3.94	1.70		0.03		weak	
Total OCD severity score	7.78ᵇ	6.47, 9.09	6.17ᵃ	5.44, 6.90	4.29		0.07		weak	
Premonitory urges severity, PUTS¹	17.9	17.1, 18.8	17.6	17.1, 18.0	0.53		—³		weak	

Data are presented as marginal means followed by 95% confidence intervals (95% CI). Statistically significant marginal mean rankings are marked by Latin letters, with “a” indicating the smallest value in each row

RESI: Robust Effect Size Index (Vandekar, Tao, & Blume, 2020) [32] derived from the Wald χ² statistic and reported on a Cohen’s-d-like scale. Magnitude thresholds follow Vandekar et al. (2020): “weak” < 0.1, “small” 0.1–0.25, “medium” 0.25–0.4, “large” > 0.4

Abbreviations: *ADHD* Attention Deficit Hyperactivity Disorder, *OCD* Obsessive Compulsive Disorder, *ODD* Oppositional Defiant Disorder, *PUTS* Premonitory Urge for Tics Scale

¹*YGTSS* Yale Global Tic Severity Scale, *SNAP-IV* Swanson, Nolan and Pelham-IV rating scale, *CY-BOCS* Children’s Yale-Brown Obsessive Compulsive Scale, *PUTS* Premonitory Urge for Tics Scale

²Using medications with psychotropic activity other than tic-suppressing medications, or no medication use

³RESI computed to effectively zero for this non-significant comparison

### The severity scores of tics and comorbid psychopathology among different medication groups

**Fig. 2:**
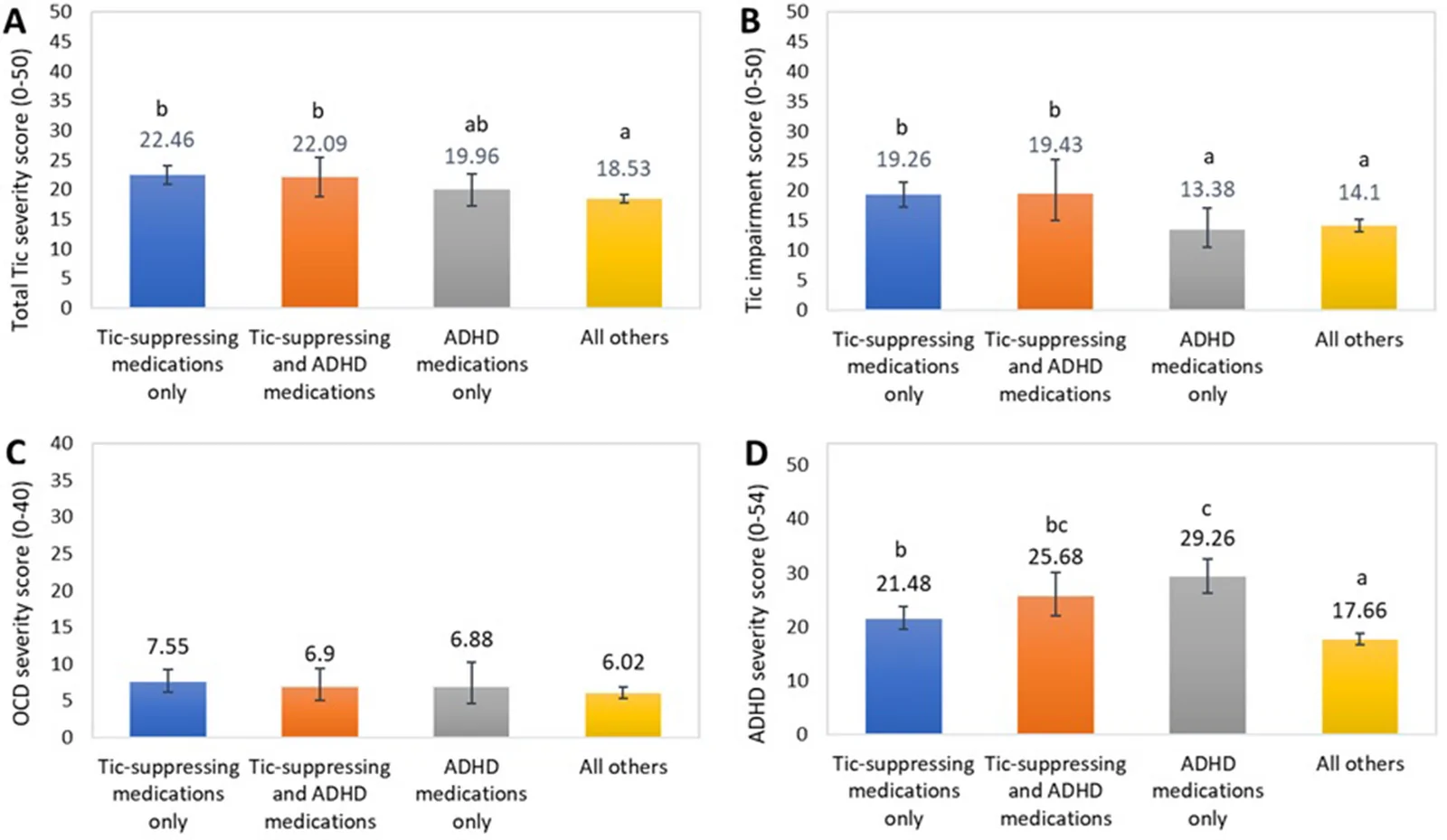
Symptom Severity Across Four Medication Subgroups. This figure compares scores between four different groups of children and adolescents, based on what kind of medications they were receiving: Tic-suppressing medications only, Tic-suppressing and ADHD medications, Non tic-suppressing medications and All others (i.e., children and adolescents who either took other psychotropic medications or none at all). (**A**) Total tic severity score (YGTSS), (**B**) Tic impairment score (YGTSS), (**C**) OCD severity score (CY-BOCS), and (**D**) ADHD severity score (SNAP-IV). Data are presented as a mean (average), with 95% confidence intervals (CI). YGTSS, Yale Global Tic Severity Scale; CY-BOCS, Children’s Yale-Brown Obsessive-Compulsive Scale; SNAP-IV, Swanson, Nolan, and Pelham Questionnaire

Table 4; Fig. 2 show the association of treatment grouping (i.e., tic-suppressing medications only, tic-suppressing and ADHD medications, ADHD medications only and all others [i.e., those using no or other types of psychotropic medications] with symptom severity scores. These differed across the four medication groups in all tic measures (severity and impairment), and ADHD and ODD symptoms, but not in OCD symptoms and the premonitory urges. Participants treated with tic-suppressing medication, with or without ADHD medication, exhibited higher levels of tic severity compared predominantly to “all others” (Table 4; Fig. 2). Participants receiving tic-suppressing medications, with or without ADHD medications, reported significantly higher levels of tic-related impairment compared to participants with no tic-suppressing treatment (i.e., either ADHD medications alone or else). The ADHD medications-only group showed the highest ADHD and ODD symptom levels related to ADHD and ODD, followed by the group using both tic-suppressing and ADHD medications.

**Table 4:** Severity Scores of Tics and Comorbid Psychopathology across Medication Groups (*N* = 709, Covariate-Adjusted Generalized Linear Models)

Symptoms and comorbidities	Tic-suppressing medications only (Tic only)*n* = 141, 19.9%		Tic-suppressing medications + ADHD (Tic+ADHD)*n* = 34, 4.8%		ADHD medications only (ADHD only)*n* = 39, 5.5%		All others² (All) *n* = 495, 69.8%		Wald’s χ²	RESI	Magnitude
	Mean	95% CI	Mean	95% CI	Mean	95% CI	Mean	95% CI			
Tics (YGTSS¹)											
Motor tics	13.7ᶜ	12.9, 14.4	13.9ᵇᶜ	12.1, 15.6	12.2ᵃᵇ	11.0, 13.4	11.8ᵃ	11.4, 12.2	20.76	0.17	small
	Tic only = Tic+ADHD > All; Tic+ADHD = ADHD only; Tic only > ADHD only = All										
Vocal tics	8.81ᵇ	7.78, 9.84	8.22ᵃᵇ	6.32, 10.12	7.83ᵃᵇ	5.95, 9.72	6.72ᵃ	6.25, 7.19	13.87	0.13	small
	Tic only > All; Tic only = Tic+ADHD = ADHD only; Tic+ADHD = ADHD only = All										
Total tic severity	22.5ᵇ	20.9, 24.0	22.1ᵇ	18.8, 25.4	20.0ᵃᵇ	17.2, 22.7	18.53ᵃ	17.8, 19.2	21.93	0.17	small
	Tic only = Tic+ADHD > All; Tic only = Tic+ADHD = ADHD only; ADHD only = All										
Tic impairment	18.7ᵇ	16.6, 20.8	18.7ᵇ	13.6, 23.7	12.5ᵃ	9.30, 15.8	13.2ᵃ	12.3, 14.2	24.69	0.18	small
	Tic only = Tic+ADHD > ADHD only = All										
ADHD symptoms (SNAP-IV¹)											
Inattention	11.9ᵇ	10.8, 13.0	14.6ᶜ	12.4, 16.9	16.1ᶜ	14.2, 17.9	9.59ᵃ	9.01, 10.2	59.70	0.29	medium
	Tic+ADHD = ADHD only > Tic only > All										
Hyperactivity	9.51ᵇ	8.43, 10.6	11.1ᵇᶜ	9.05, 13.1	13.3ᶜ	11.3, 15.4	8.24ᵃ	7.70, 8.78	28.07	0.20	small
	ADHD only > Tic only > All; ADHD only = Tic+ADHD; Tic + ADHD = Tic only										
Combined	21.5ᵇ	19.5, 23.7	25.8ᵇᶜ	21.9, 29.8	29.4ᶜ	26.3, 32.5	17.8ᵃ	16.8, 18.8	61.08	0.29	medium
	ADHD only > Tic only > All; Tic+ADHD = ADHD only; Tic+ADHD = Tic only										
ODD symptoms (SNAP-IV ODD¹)	9.05ᵇ	7.99, 10.1	8.54ᵃᵇ	6.75, 10.3	10.1ᵇ	8.44, 11.8	7.45ᵃ	6.95, 7.95	14.52	0.14	small
	Tic only = ADHD only > All; Tic+ADHD = All; Tic+ADHD = Tic only; Tic+ADHD = ADHD only										
OCD symptoms (CY-BOCS¹)											
Obsessions	3.61	2.75, 4.47	3.47	1.88, 5.06	2.45	1.12, 3.77	2.72	2.33, 3.12	4.33	0.07	weak
Compulsions	4.19	3.38, 5.20	3.59	2.39, 5.38	3.71	2.49, 5.52	3.50	3.10, 3.95	2.11	0.04	weak
Total OCD severity score	7.55	6.13, 9.29	6.90	5.08, 9.37	6.88	4.60, 10.3	6.02	5.34, 6.78	4.39	0.07	weak
Premonitory urges severity, PUTS¹	17.8	16.9, 18.8	17.8	16.1, 19.6	15.4	13.9, 17.2	17.7	17.2, 18.2	6.24	0.09	weak

Data are presented as marginal means followed by 95% confidence intervals (95% CI). Statistically significant marginal mean rankings are marked by Latin letters, with “a” indicating the smallest value in each row

RESI: Robust Effect Size Index (Vandekar, Tao, & Blume, 2020), derived from the Wald χ² statistic and reported on a Cohen’s-d-like scale. Magnitude thresholds follow Vandekar et al. (2020): “weak” < 0.1, “small” 0.1–0.25, “medium” 0.25–0.4, “large” > 0.4

Abbreviations: *ADHD* Attention Deficit Hyperactivity Disorder, *OCD* Obsessive Compulsive Disorder, *ODD* Oppositional Defiant Disorder, *PUTS* Premonitory Urge for Tics Scale

¹*YGTSS* Yale Global Tic Severity Scale, *SNAP-IV* Swanson, Nolan and Pelham-IV rating scale, *CY-BOCS* Children’s Yale-Brown Obsessive Compulsive Scale, *PUTS* Premonitory Urge for Tics Scale

²Using medications with psychotropic activity other than tic-suppressing or ADHD medications, or no medication use

### Supplementary

Among the control variables (covariates), age showed a positive association on most of the continuous severity symptoms, indicating that older subjects exhibited higher symptom levels compared to younger ones. Sex differences were found in levels of motor tics, hyperactivity, and combined ADHD symptoms, with girls showing lower levels, on average. Similarly, higher education was associated with lower symptoms in vocal tics, tic severity, and ADHD symptoms. Complete model results can be found in the supplementary materials (Table S2 and Table S3).

## Discussion

To our knowledge, this is the first study to characterize pharmacological treatment of pediatric chronic tic disorders in a harmonized, multi-country European clinical cohort. Our findings extend the existing literature by linking medication groups directly to clinician-rated symptom severity in the same individuals, by documenting substantial cross-national variability in real-world prescribing across nine European countries, and by identifying a consistent under-representation of alpha-2 agonists relative to guideline recommendations. Our study confirms that medication use in youth with CTD is associated with higher tic severity and greater impairment due to tics across all tics and ADHD evaluated scores and in some of the OCD scores. As expected, youth with comorbid ADHD more often received medication, predominantly ADHD medications. Notably, participants with OCD but no ADHD received a similar proportion of tic-suppressing medications compared to those without any of these comorbidities. We found that medication use was higher in adolescents than in children with or without OCD, but not in those with ADHD [16].

Approximately a third of the participants in this study were treated with pharmacological interventions at baseline. This prevalence rate is comparable to the use of any psychotropic drug (38.3%) reported among newly diagnosed children in a nationwide Danish cohort (2014–2017) [10], in a comparable time period to the EMTICS study, but is notably lower than the 64% prevalence found in a UK specialist Tourette syndrome clinic cohort, which included serial pharmacological trials [34, 35]. However, findings across these studies consistently show that medication use is associated with greater symptom severity and the presence of comorbidities; the medicated patients in the UK specialist clinic cohort (which included mostly adult patients, with limited data from children) also had significantly higher baseline tic severity scores (YGTSS mean = 26.5) than the non-medicated group (YGTSS mean = 21.8)^34^. Also in line with our findings, several studies have shown that patients receiving tic-suppressing medications generally present with higher tic severity, reflecting the clinical practice of reserving pharmacotherapy for more impairing cases [10, 35, 36].

The type of medication usage among the study subjects was consistent with the literature [10]. Most of the participants with pharmacological treatment were treated with antipsychotic drugs, mainly second-generation antipsychotics. Following in prevalence were ADHD medications, with only a minority treated with alpha-2-agonists. It is worth noting that 18 participants (25%) who were treated with ADHD medications did not have a formal ADHD diagnosis. At the same time, according to European guidelines, antipsychotics are indicated for severe tics and coexisting OCD, while alpha-2 agonists (noradrenergic agents like clonidine and guanfacine) are recommended for subjects with tics and comorbid ADHD [7]. Our study shows that alpha-2 adrenergic agonists remain infrequently prescribed, even among participants with ADHD (9/195), despite established efficacy and a favorable side effect profile [37]. In North America, they are considered a first-line treatment for tics, particularly in the context of comorbid ADHD [11, 12], whereas European clinicians more often favor antipsychotics [6, 38–41]. This observed preference likely results from a lack of approval of guanfacine for the treatment of ADHD during the data collection period (2013–2015). At that time, guanfacine was not yet approved for tic disorders or ADHD in European countries. Clonidine, although available in Europe for decades for the treatment of hypertension, has likewise not been formally approved for pediatric tic disorders or ADHD in most European countries, leaving its use in this population off-label. In practice, antipsychotics are typically prioritized for more severe tics, while alpha-2-adrenergic agonists are reserved for adjunctive or secondary use, despite well-established benefits for ADHD [42]. Additionally, the limited number of FDA and European approvals for tic-suppressing medications results in widespread off-label prescribing, underscoring the need for additional clinical trials to support regulatory approval.

The country-level distribution of alpha-2 agonist use is striking. Across the EMTICS baseline cohort (*N* = 709), only 20 participants (2.8%) received any alpha-2 agonist. Three countries with substantial cohorts, Germany (*n* = 123), Hungary (*n* = 80), and Switzerland (*n* = 39), comprising 242 patients (34.1% of the total sample) recorded zero alpha-2 prescriptions during the 2013–2015 study window. Among the remaining six countries, prescribing was sparse, with the highest rates observed in Britain (7/60, 11.7%) and Denmark (3/24, 12.5%). The remaining countries showed minimal use (Israel 3/65, 4.6%; Netherlands 3/74, 4.1%; Italy 3/166, 1.8%; Spain 1/78, 1.3%). This under-representation of alpha-2 agonists in EMTICS may be attributed to variable factors including European authorization of guanfacine occurred only after the study period (2015), the absence of a pediatric tic indication for clonidine in most participating countries, formulary and reimbursement variability, and uneven prescriber familiarity. Concerns about sedation, orthostatic hypotension, and the risk of rebound hypertension with abrupt discontinuation are commonly cited barriers, and may discourage initial prescribing even where regulatory access is available [42]. Additionally, training traditions differ across the prescriber specialties involved in pediatric tic care (child psychiatry, pediatric neurology, developmental pediatrics)[39], and pediatric-appropriate formulations and dosing guidance for alpha-2 agonists in tic disorders were less widely disseminated during the study period than they are today, factors that together may have further contributed to the observed cross-national heterogeneity in treatment.

Our findings indicate that the presence of comorbid ADHD, either alone or alongside OCD, is associated with a more than twofold increase in the likelihood of receiving any type of pharmacological treatment, yet largely explained by ADHD medication, and accompanied by a lower use of tic-suppressing medications, compared to individuals with CTD alone or CTD with comorbid OCD. This is consistent with prior research demonstrating higher medication use in subjects with tics and ADHD comorbidity [1, 34, 43, 44], likely reflecting the greater functional impairment, high internalizing symptoms and clinical urgency typically associated with attentional difficulties [43–45]. Reduced use of tic-suppressing agents in the presence of ADHD, but not OCD, suggests that clinical attention may shift toward managing ADHD symptoms when both conditions coexist.

Although this cross-sectional study cannot examine the impact of medications, the finding that children receiving tic-suppressing medication, whether alone or combined with ADHD medication, have more severe tics reflects clinical prescribing patterns where medications are reserved for more severe cases. This highlights the association between higher symptom load and the likelihood of receiving pharmacological treatment. According to ESSTS guidelines, an average tic reduction of 50% can be expected; however, individual responses vary widely, with some patients reporting up to 90% improvement, while others experience little to no benefit [7]. Notably, participants receiving tic-suppressing medications reported significantly higher levels of tic-related impairment compared to participants receiving non-tic-suppressing medications or no treatment at all. Greater impairment indicates a greater subjective impact of tics on participants’ lives. Previous research suggests that functional impairment, more than symptom severity, is strongly linked to the likelihood of receiving pharmacological treatment for tic disorders [46].

Clinical guidelines from both the Centers for Disease Control and Preventions (CDC) and ESSTS, emphasize that treatment is recommended when tics impair school, social, or emotional functioning, and clinicians are advised to assess tic-related impairment as part of decision-making [46–48]. The current study also highlights the key role of parental perception in treatment choices, particularly when caregivers believe tics negatively affect the child’s quality of life. These subjective judgments often drive clinical action, underscoring the importance of thorough psychoeducation and functional assessment during care planning. Behavioral interventions and psychoeducation cannot only aid in symptom management but also improve family understanding, strengthen coping strategies, and enhance resilience.

In this study, children and adolescents with chronic tics treated exclusively with ADHD medication showed more severe ADHD and ODD symptoms, as may be expected. Similarly, those receiving combined treatment for both ADHD and tics also exhibited elevated levels of these comorbid symptoms. In contrast, participants treated solely with tic-suppressing medications or other psychotropic agents showed lower scores of ADHD and ODD symptoms. These findings suggest that pharmacological treatment is generally aligned with clinical need, as it is more frequently prescribed to individuals with more severe comorbid symptoms like ADHD/ODD.

Lastly, the current study found that participants receiving medication solely for ADHD and no tic-suppressing medication had lower tic severity symptoms compared to other treatment groups. This is in line with expectations given that tic-suppressing medication is recommended for more severely affected cases. Also, no significant difference in tic severity was observed between those treated only with tic-suppressants and those receiving combined treatment with ADHD medications. These findings show that stimulant use was not associated with higher tic severity in our cohort, consistent with previous reports suggesting that stimulants may not necessarily worsen tics at the group level. In fact, consistent with our results, recent studies suggest that stimulants do not increase tic severity and may even lead to moderate improvement, possibly by enhancing self-regulatory control [7, 41, 49].

A particularly notable finding of this study is that premonitory urge intensity, as measured by the PUTS, did not differ significantly between participants receiving tic-suppressing medications and those not receiving them (Tables 3 and 4), despite the medicated groups showing substantially higher motor and total tic severity and greater tic-related impairment. Few explanations merit consideration. First, the pharmacological action of antipsychotics, the dominant class of tic-suppressing agents in our cohort, primarily attenuates motor output of the tic loop via D2 receptor modulation, and may have limited effect on the sensory phenomenology of premonitory urge generation itself. This is consistent with patient reports that medications reduce tic frequency without abolishing urges [13, 36]. Second, premonitory urge awareness is developmentally acquired and is typically not reliably reported by younger children; given a cohort mean age of 10.7 years, the measure is subject to age-related reporting variability that may attenuate between-group differences [3, 28]. Third, the PUTS is a self-report measure of a subjective experience, and its sensitivity to medication-related change has not been comprehensively characterized; ceiling, floor, and content-validity considerations may limit cross-sectional between-group resolution [3, 29]. The clinical implication is that pharmacological tic-suppression should not be expected to eliminate premonitory urges, and that patients and families should be counseled accordingly. The finding also supports the continued importance of behavioral interventions targeting the urge-generation circuitry directly, including habit reversal training and exposure-based therapies, as essential complements to pharmacological treatment, regardless of medication response on the motor side [47].

### Limitations and future research

Several limitations and considerations should be mentioned. As this study includes cross-sectional data collected between 2013–2015, our findings may not reflect current prescribing trends or shifts in medication use (see e.g., Rasmussen et al., 2020) [10]. Participants were recruited from clinical settings or patient organizations possibly having received prior treatment. Therefore, the observed associations are subject to confounding by indication and should not be interpreted as medication effects. Rather, they reflect real-world prescribing patterns in relation to symptom severity. Our study lacked systematic documentation on symptom severity before treatment initiation, which limited our ability to draw conclusions about adherence to treatment recommendations for TS/CTD and related disorders. Also, the specific indication for each prescribed medication could not be determined (for example, antipsychotic drug use may have been prescribed for tic suppression, behavioral disorders, and/or for psychotic or affective disorders). We categorized alpha-2 adrenergic agonists (clonidine, guanfacine) as tic-suppressing rather than non-stimulant ADHD medications, consistent with prior guidelines and practices. Guanfacine was only authorized in Europe as of 2015, after the study started, and is therefore underrepresented. Clonidine, although available in Europe for decades for other indications, has not been formally approved for pediatric tic disorders or ADHD in most European countries during the study period; its use in this population was therefore off-label and similarly low.

A particularly important limitation is the absence of systematically recorded behavioral therapy data. Behavioral interventions, including habit reversal training (HRT) and the broader Comprehensive Behavioral Intervention for Tics (CBIT) protocol, as well as exposure and response prevention (ERP), are first-line recommended treatments for tic disorders according to ESSTS guidelines [47], and prior or concurrent behavioral treatment is a likely major determinant of pharmacological decision-making in real-world practice [9]. Future cohorts should incorporate structured documentation of behavioral interventions alongside pharmacological data.

Furthermore, selection bias may have affected comparability with other studies. For example, children with ADHD may have been less likely to participate due to high study demands. Our cohort included 27.5% youth with comorbid ADHD, which is lower than rates often reported in specialized clinical samples (~ 40–50%)48, but higher than some administrative registry estimates (e.g., ~ 11–15% in German claims data)49. Finally, although ASSQ-based screening for autistic traits is included in Table 1 to characterize the cohort’s clinical complexity, ASD was not used as an analytic stratifier in this study. This is because the ASSQ is a screening questionnaire for autistic traits rather than a diagnostic instrument, and clinician-confirmed ASD diagnoses suitable for analytic stratification were not available in the EMTICS baseline protocol. We therefore did not target ASD comorbidity in our analyses.

Future systematic large-scale pharmacoepidemiologic studies, such as nationwide register studies, are needed to provide more insight into current treatment patterns and trends across age and sex/gender. Future work should evaluate longitudinal treatment trajectories (switching or discontinuation) and relate them to response and tolerability, and evaluate drug effectiveness, compare different pharmacological options, and examine their impact on comorbid conditions associated with tic disorders.

In summary, our findings show that about one-third of the study participants received pharmacological treatment and that medication use was associated with higher symptom severity scores and greater impairment across all tics and ADHD evaluated scores and in some of the OCD scores. Consistent with guideline recommendations, the most commonly used classes were antipsychotics (mostly second generation) and ADHD medications (mostly stimulants), however, alpha-2 adrenergic agonist use was limited. These findings provide an empirical reference point against which subsequent prescribing trends and guideline-uptake initiatives can be evaluated, and point to the need for integrated treatment approaches that incorporate behavioral and psychoeducational interventions, underscoring the importance of developing personalized treatment plans that combine multiple strategies.

## Supplementary Information

Below is the link to the electronic supplementary material.

Supplementary Material 1 (DOCX 45.0 KB)

## Data Availability

No datasets were generated or analysed during the current study.

## Abbreviations

ADHD: Attention Deficit Hyperactive Disorder
OCD: Obsessive Compulsive Disorder
ODD: Oppositional Defiant Disorder
PUTS: Premonitory Urge for Tics Scale
