# Supplementary material for: Medication use and treatment patterns in children and adolescents with chronic tic disorders: an EMTICS study
Source: Eur Child Adolesc Psychiatry. 2026 Jul 17;35(11):3657–72. doi: 10.1007/s00787-026-03120-5 (PMC13710581; doi:10.1007/s00787-026-03120-5)
Supplement: Supplementary file 1 [file 787_2026_3120_MOESM1_ESM.docx]

**Supplementary Information**

**Medication Use and Treatment Patterns in Children and Adolescents with Chronic Tic Disorders: An EMTICS Study**

Orit Peled^1,2^, Daphna Shoham^3,4^, Tamar Steinberg^4,5^, Alan Apter^3,4^, Dana Feldman-Sadeh^3,4^, Miri Carmel^4,6^, Idan Barak^3^, Zsanett Tarnok^7^, Peter Nagy^7,8^, Abraham Weizman^4,6^, Pieter J. Hoekstra^9,10^, Andrea Dietrich^9,10^, the EMTICS collaborative group, Noa Benaroya-Milshtein^3,4^

^1^Department of Pharmacy, Schneider Children’s Medical Center of Israel, Petah Tikvah, Israel.

^2^Adelson School of Medicine, Ariel University, Ariel, Israel.

^3^Department of Child and Adolescent Psychiatry, Schneider Children's Medical Center of Israel, Petah Tikvah 4920235, Israel.

^4^Gray Faculty of Medical & Health Sciences, Tel Aviv University, Israel.

^5^Institute of Child Neurology, Schneider Children's Medical Center of Israel, Petach Tikva, Israel.

^6^Research Unit, Geha Mental Health Center, and Laboratory of Biological Psychiatry, Felsenstein Medical Research Center, Petah Tikvah, Israel.

^7^Vadaskert Child and Adolescent Psychiatric Hospital, Budapest, Hungary.

^8^Pediatrics Clinic of Semmelweis University, Semmelweis University, Budapest, Hungary.

^9^Department of Psychiatry, University Medical Center Groningen, University of Groningen, Groningen, The Netherlands.

^10^Accare Child Study Center, Groningen, The Netherlands.

**Corresponding author**: Noa Benaroya-Milshtein, Department of Child and Adolescent Psychiatry, Schneider Children's Medical Center of Israel, Petach Tikva 4920235, Israel; Faculty of Medicine, Tel Aviv University, Tel Aviv, Israel

Tel: 972-3-9253616 Fax 972-3-9253864; Email: [nbenaroya@gmail.com](mailto:nbenaroya@gmail.com), [noabena@clalit.org.il](mailto:noabena@clalit.org.il)

**Table of contents**

**S1**. Classification of Current Psychotropic Medications by Type

**Table S2.** Severity Scores of Tic-Related Symptoms and Comorbid Psychopathology among Children and Adolescents with or without Tic-Suppressing Medications controlled by main personal characteristic variables (N=709)

**Table S3.** Severity Scores of Tic-Related Symptoms and Comorbid Psychopathology across Medication Groups controlled by main personal characteristic variables (N=709)

**Table S4.** Effect sizes (Cohen's h) for pairwise proportion comparisons in Table 2

**S1**. Classification of Psychotropic Medications by Type

**Antipsychotics**

**First generation Antipsychotics**

Pimozide

Haloperidol

Fluphenazine

Periciazine

**Second generation Antipsychotics**

Aripiprazole

Olanzapine

Quetiapine

Risperidone

Tiapride

Sulpiride

Amisulpride

**ADHD**

**Stimulants**

Methylphenidate

Amphetamines

Dexamphetamine

Lisdexamphetamine

Mixed amphetamine salts

**Non-stimulants**

Atomoxetine

**Alpha Agonists**

Clonidine

Guanfacine

**Antidepressants**

**SRIs**

Citalopram

Escitalopram

Fluoxetine

Fluvoxamine

Paroxetine

Sertraline

Venlafaxine

**Other**

Nortriptyline

Mirtazapine

Clomipramine

Doxepine

**Benzodiazepines**

Clonazepam

Alprazolam

Lorazepam

Clorazepate

Lormetazepam

Diazepam

Oxazepam

**Other**

Cannabis

Propranolol

*Abbreviations: ADHD,* *Attention Deficit Hyperactive Disorder; SRI, Serotonin Reuptake Inhibitors.*

**Tic-suppressing medications** were defined as first- and second-generation antipsychotics and included α2-adrenergic agonists (e.g., clonidine), which are recommended for both tic reduction and the treatment of ADHD. Note: guanfacine was not included in the predefined medication listing.

**ADHD medications** comprised agents indicated exclusively for ADHD, including stimulant and non-stimulant formulations.

**Table S2.** Severity Scores of Tic-Related Symptoms and Comorbid Psychopathology among Children and Adolescents with or without Tic-Suppressing Medications controlled by main personal characteristic variables (N=709)

|  |  | Tic Medications  Mean [95% CI] | Other^##^  (Non Tic Medications)  Mean [95% CI] | Group | Age | Sex | ADHD Medication | Education | Country | Ethnicity |
| --- | --- | --- | --- | --- | --- | --- | --- | --- | --- | --- |
|  | **Tics** (YGTSS^#^) |  |  |  |  |  |  |  |  |  |
| N | Motor tics | 13.7^b^  [13.0, 14.4] | 11.8^a^  [11.5, 12.2] | 1.86***  (0.41) | 0.15**  (0.06) | -0.91*  (0.40) | 0.40  (0.57) | -0.06  (0.15) | -0.14  (0.37) | 1.45**  (0.48) |
| 2P | Vocal tics |  |  |  |  |  |  |  |  |  |
|  | Continuous | 11.4^b^  [9.0,13.7] | 10.0^a^  [7.9,12.2] | 1.37**  (0.42) | 0.13*  (0.07) | -0.33  (0.43) | 0.40  (0.59) | -0.45**  (0.16) | -0.69  (0.41) | -0.02  (0.49) |
|  | Binary | 0.20^b^  [0.02,0.36] | 0.14^a^  [0.01,0.27] | 0.44*  (0.23) | -0.02  (0.03) | -0.10  (0.20) | 0.03  (0.31) | -0.09  (0.08) | -0.11  (0.20) | -0.32  (0.26) |
|  |  |  |  |  |  |  |  |  |  |  |
| N | Total tic severity | 22.4^b^  [21.1, 23.7] | 18.6^a^  [17.9, 19.4] | 3.74***  (0.76) | 0.23*  (0.11) | -1.33  (0.75) | 0.86  (1.06) | -0.58*  (0.29) | -0.75  (0.69) | 0.95  (0.91) |
| G | Tic impairment | 19.3^b^  [17.5, 21.4] | 14.0^a^  [13.2, 15.0] | 0.32***  (0.06) | 0.04***  (0.01) | -0.06  [0.07) | -0.34  (0.10) | -0.04  (0.03) | 0.06  (0.07) | 0.01  (0.11) |
|  | **ADHD** **symptoms** (SNAP-IV^#^) |  |  |  |  |  |  |  |  |  |
| N | Inattention | 11.9^b^  [10.9, 12.9] | 10.3^a^  [9.9, 10.8] | 1.65**  (0.60) | 0.24**  (0.09) | -0.89  (0.59) | 4.37***  (0.84) | -0.50*  (0.22) | 1.87*  (0.83) | -1.01  (0.71) |
| G | Hyperactivity | 9.7^b^  [8.7, 10.7] | 8.46^a^  [7.95, 9.00] | 0.13*  (0.06) | -0.03**  (0.01) | -0.18**  (0.07) | 0.39***  (0.07) | -0.08**  (0.02) | -0.03  (0.06) | -0.09  (0.07) |
| G | Combined | 21.6^b^  [19.8, 23.6] | 18.51^a^  [17.6, 19.5] | 0.16**  (0.05) | 0.003  (0.01) | -0.14*  (0.06) | 0.40***  (0.06) | -0.06**  (0.02) | -0.09  (0.06) | -0.06  (0.07) |
| G | **ODD** **symptoms** (SNAP-IV ODD^#^) | 8.87^b^  [7.65, 9.75] | 7.7^a^  [7.19, 8.18] | 1.18*  (0.56) | -0.11  (0.08) | -0.69  (0.51) | 1.26  (0.73) | -0.71***  (0.19) | 0.30  (0.53) | -1.49*  (0.62) |
| G | **OCD** **symptoms** (CY-BOCS^#^) |  |  |  |  |  |  |  |  |  |
| G | Obsessions | 3.41^b^  [2.73, 4.26] | 2.56^a^  [2.22, 2.96] | 0.29*  (0.14) | 0.04  (0.02) | 0.06  (0.14) | 0.13  (0.19) | -0.05  (0.06) | 0.77***  (0.13) | -0.48**  (0.17) |
| G | Compulsions | 4.02  [3.30, 4.89] | 3.52  [3.13, 3.95] | 0.13  (0.12) | 0.06**  (0.02) | -0.17  (0.13) | -0.06  (016) | -0.08  (0.05) | -0.14  (0.11) | 0.01  (0.15) |
| G | Total OCD severity score | 7.59  [6.30, 9.14] | 6.14  [5.49, 6.87] | 0.21  (0.11) | 0.05**  (0.02) | -0.07  (0.14) | -0.08  (0.14) | -0.08  (0.04) | -0.002  (0.11) | -0.13  (0.15) |
| G | **Premonitory urges severity** (PUTS^#^) | 18.0  [17.1, 18.8] | 17.5  [17.0, 18.0] | 0.03  (0.03) | 0.03***  (0.004) | 0.03  (0.03) | -0.07  (0.04) | 0.02  (0.01) | - | -0.04  (0.03) |

*** p<.001, ** p<.01, * p<.05; Standard errors in parentheses; 95% confidence interval in squared brackets;

Distribution: N Normal; G Gamma; 2P Two Parts. Statistically significant marginal mean rankings are marked by Latin letters, with "a" indicating the smallest value in each row.

^#^*YGTSS – Yale Global Tic Severity Scale; SNAP-IV – Swanson, Nolan and Pelham-* *IV rating scale; CYBOCS – Children’s Yale-Brown Obsessive Compulsive Scale; PUTS -* *Premonitory Urge for Tics Scale, using clinical cut-off score >9 for diagnosis;*

^##^Using medications with psychotropic activity other than tic-suppressing medications or no medication use.

*Abbreviations:* *ADHD, Attention Deficit Hyperactive Disorder; OCD, Obsessive Compulsive Disorder; ODD, Oppositional Defiant Disorder; PUTS,* *Premonitory Urge for Tics Scale;*

**Table S3.** Severity Scores of Tic-Related Symptoms and Comorbid Psychopathology across Medication Groups controlled by main personal characteristic variables (N=709)

|  |  | Tic Medications only  Mean  [95% CI] | Tic + ADHD Medications  Mean  [95% CI] | ADHD Medications only  Mean  [95% CI] | Other^##^ (non of previous)  Mean  [95% CI] | Group | Age | Sex | Education | Country | Ethnicity |
| --- | --- | --- | --- | --- | --- | --- | --- | --- | --- | --- | --- |
|  | **Tics** (YGTSS^#^) |  |  |  |  |  |  |  |  |  |  |
| N | Motor tics | 13.7^c^  [12.9, 14.4] | 13.9^bc^  [12.1. 15.6] | 12.2^ab^  [11.01, 13.36] | 11.8^a^  [11.4, 12.2] | Wald=  20.8*** | 0.15*  (0.06) | -0.90*  (0.40) | -0.06  (0.15) | -0.31  (0.35) | 1.39**  (0.54) |
| 2P | Vocal tics |  |  |  |  |  |  |  |  |  |  |
|  | Continuous | 11.3^b^  [9.0, 13.9] | 11.0^ab^  [8.5, 12.7] | 10.9^ab^  [8.3, 13.7] | 9.73^a^  [7.79, 12.0] | Wald^1^=  11.5** | 0.13*  (0.07) | -0.30  (0.44) | -0.39*  (0.15) | -0.37  (0.36) | -0.10  (0.53) |
|  | Binary | .20^a^  [.07, .45] | .17^a^  [.05, .52] | .15^a^  [.04, .43] | .13^a^  [.05, .30] | Wald^1^=  5.90 | -0.02  (0.03) | -0.09  (0.21) | -0.08  (0.08) | -0.20  (0.17) | -0.32  (0.28) |
| N | Total tic severity | 22.7^b^  [20.9, 24.0] | 22.1^b^  [18.8, 25.4] | 20.0^ab^  [17.2, 22.7] | 18.5^a^  [17.8, 19.2] | Wald=  22.0*** | 0.24*  (0.12) | -1.23  (0.73) | -0.47  (0.28) | -1.53*  (0.67) | 1.08  (0.96) |
| G | Tic impairment | 19.2^b^  [17.3, 21.4] | 19.4^b^  [14.9, 25.9] | 13.4^a^  [10.5, 17.0] | 14.1^a^  [13.2, 15.1] | Wald=  28.2*** | 0.04***  (0.01) | -0.06  (0.07) | -0.05  (0.03) | 0.10  (0.08) | 0.06  (0.10) |
|  | **ADHD** **symptoms**, (SNAP-IV^#^) |  |  |  |  |  |  |  |  |  |  |
| N | Inattention | 11.9^b^  [10.8, 13.0] | 14.6^c^  [12.4, 16.8] | 16.1^c^  [14.0, 18.2] | 9.6^a^  [9.0, 10.2] | Wald=  54.1*** | 0.26**  (0.09) | -1.06  (0.59) | -0.53*  (0.22) | -0.93  (0.50) | -0.97  (0.70) |
| G | Hyperactivity | 9.4^b^  [8.4, 10.6] | 10.9^bc^  [9.1, 13.2] | 13.4^c^  [11.4, 15.7] | 8.1^a^  [7.6, 8.7] | Wald=  36.8*** | -0.03**  (0.01) | -0.21**  (0.07) | -0.08**  (0.02) | 0.20**  (0.06) | -0.15*  (0.07) |
| G | Combined | 21.5^b^  [19.5, 23.7] | 25.7^bc^  [22.0, 30.0] | 29.3^c^  [26.3, 32.6] | 17.7^a^  [16.7, 18.7] | Wald=  75.2*** | 0.00  (0.01) | -0.14*  (0.06) | -0.06**  (0.02) | 0.06  (0.16) | -0.09  (0.06) |
| G | **ODD** **symptoms**, (SNAP-IV ODD^#^) | 8.93^b^  [7.92, 10.07] | 8.57^ab^  [7.01, 10.5] | 10.0^b^  [8.5, 11.9] | 7.40^a^  [6.91, 7.92] | Wald=  15.3** | -0.02  (0.01) | -0.09  (0.07) | -0.09***  (0.02) | -0.07  (0.06) | -0.19*  (0.07) |
| G | **OCD** **symptoms,** (CY-BOCS^#^) |  |  |  |  |  |  |  |  |  |  |
| G | Obsessions | 3.62  [2.84, 4.62] | 3.65  [2.37, 5.64] | 2.84  [1.67, 4.83] | 2.60  [2.24, 3.01] | Wald=  5.87 | 0.04*  (0.02) | -0.03  (0.14) | -0.13*  (0.05) | 0.50***  (0.15) | -0.51**  (0.18) |
| G | Compulsions | 4.19  [3.38, 5.20] | 3.59  [2.39, 5.38] | 3.71  [2.49, 5.52] | 3.50  [3.10, 3.95] | Wald=  2.06 | 0.05**  (0.02) | -0.17  (0.13) | -0.06  (0.05) | 0.05  (0.11) | -0.05  (0.15) |
| G | Total OCD severity score | 7.55  [6.13, 9.29] | 6.90  [5.08, 9.37] | 6.88  [4.60, 10.3] | 6.02  [5.34, 6.78] | Wald=  3.72 | 0.05**  (0.02) | -0.14  (0.12) | -0.08  (0.04) | 0.35**  (0.11) | -0.22  (0.15) |
| G | **Premonitory urges severity** (PUTS^#^) | 17.8  [16.9, 18.8] | 17.8  [16.1, 19.6] | 15.4  [13.9, 17.2] | 17.7  [17.2, 18.2] | Wald=  6.22 | 0.03***  (0.01) | 0.03  (0.03) | 0.01  (0.01) | 0.02  (0.03) | -0.04  (0.04) |

*** p<.001, ** p<.01, * p<.05; Standard errors in parentheses; 95% confidence interval in squared brackets;

Distribution: N Normal; G Gamma; 2P Two Parts.

Statistically significant marginal mean rankings are marked by Latin letters, with "a" indicating the smallest value in each row.

^1^assessed separately within a GLM modeling framework.

^#^*YGTSS – Yale Global Tic Severity Scale; SNAP-IV – Swanson, Nolan and Pelham-* *IV rating scale; CYBOCS – Children’s Yale-Brown Obsessive Compulsive Scale; PUTS -* *Premonitory Urge for Tics Scale, using clinical cut-off score >9 for diagnosis;*

^##^Using medications with psychotropic activity other than tic-suppressing medications, ADHD medications or no medication use.

*Abbreviations:* *ADHD, Attention Deficit Hyperactive Disorder; OCD, Obsessive Compulsive Disorder; ODD, Oppositional Defiant Disorder; PUTS,* *Premonitory Urge for Tics Scale;*

**Table S4.** Effect sizes (Cohen's h) for pairwise proportion comparisons in Table 2

| # | N | nᵢ | nⱼ | pᵢ | pⱼ | Cohen's h | Magnitude |
| --- | --- | --- | --- | --- | --- | --- | --- |
| 1 | 141 | 37 | 18 | 0.262 | 0.128 | 0.315 | small |
| 2 | 141 | 37 | 26 | 0.262 | 0.184 | 0.169 | small |
| 3 | 141 | 37 | 60 | 0.262 | 0.426 | 0.295 | small |
| 4 | 141 | 18 | 26 | 0.128 | 0.184 | 0.146 | small |
| 5 | 141 | 18 | 60 | 0.128 | 0.426 | 0.610 | medium |
| 6 | 141 | 26 | 60 | 0.184 | 0.426 | 0.465 | medium |
| 7 | 39 | 3 | 15 | 0.077 | 0.385 | 0.702 | large |
| 8 | 39 | 3 | 15 | 0.077 | 0.385 | 0.702 | large |
| 9 | 39 | 3 | 6 | 0.077 | 0.154 | 0.231 | small |
| 10 | 39 | 15 | 15 | 0.385 | 0.385 | 0.000 | small |
| 11 | 39 | 15 | 6 | 0.385 | 0.207 | 0.344 | small |
| 12 | 39 | 15 | 6 | 0.385 | 0.154 | 0.471 | medium |
| 13 | 34 | 5 | 4 | 0.147 | 0.118 | 0.082 | small |
| 14 | 34 | 5 | 12 | 0.147 | 0.353 | 0.433 | medium |
| 15 | 34 | 5 | 5 | 0.147 | 0.147 | 0.000 | small |
| 16 | 34 | 4 | 12 | 0.118 | 0.353 | 0.514 | medium |
| 17 | 34 | 4 | 5 | 0.118 | 0.147 | 0.082 | small |
| 18 | 34 | 12 | 5 | 0.353 | 0.147 | 0.433 | medium |
| 19 | 229 | 50 | 46 | 0.218 | 0.201 | 0.039 | small |
| 20 | 229 | 50 | 59 | 0.218 | 0.258 | 0.083 | small |
| 21 | 229 | 50 | 75 | 0.218 | 0.328 | 0.217 | small |
| 22 | 229 | 46 | 59 | 0.201 | 0.258 | 0.121 | small |
| 23 | 229 | 46 | 75 | 0.201 | 0.328 | 0.256 | small |
| 24 | 229 | 58 | 75 | 0.253 | 0.328 | 0.143 | small |
| 25 | 230 | 76 | 50 | 0.330 | 0.217 | 0.224 | small |
| 26 | 230 | 76 | 46 | 0.330 | 0.200 | 0.972 | large |
| 27 | 230 | 76 | 58 | 0.330 | 0.252 | 0.773 | large |

*N = total sample size for the comparison group; nᵢ and nⱼ = subgroup counts; pᵢ and pⱼ = subgroup proportions; Cohen's h = effect size for the difference between two proportions (Cohen, 1988). Magnitude thresholds follow Cohen (1988): "small" h < 0.2, "small-to-medium" 0.2–0.5, "medium" 0.5–0.8, "large" ≥ 0.8.*
